# Novel Immune Features of the Systemic Inflammation Associated with Primary Hypercholesterolemia: Changes in Cytokine/Chemokine Profile, Increased Platelet and Leukocyte Activation

**DOI:** 10.3390/jcm8010018

**Published:** 2018-12-22

**Authors:** Aida Collado, Patrice Marques, Elena Domingo, Eva Perello, Herminia González-Navarro, Sergio Martinez-Hervás, José T. Real, Laura Piqueras, Juan F. Ascaso, Maria-Jesus Sanz

**Affiliations:** 1 Department of Pharmacology, Faculty of Medicine and Odontology, University of Valencia, Av. Blasco Ibáñez 15, 46010 Valencia, Spain; aida.collado@uv.es (A.C.); patricegmarques@gmail.com (P.M.); eledo2@hotmail.com (E.D.); laura.piqueras@uv.es (L.P.); 2 Department of Medicine, Faculty of Medicine and Odontology, University of Valencia, Av. Blasco Ibáñez 15, 46010 Valencia, Spain; Sergio.Martinez@uv.es (S.M.-H.); Jose.T.Real@uv.es (J.T.R.); 3 Institute of Health Research of the University Clinic Hospital of Valencia (INCLIVA), Av. Menéndez Pelayo 4, 46010 Valencia, Spain; evapc89@hotmail.com (E.P.); Herminia.Gonzalez@uv.es (H.G.-N.); 4 Diabetes and Associated Metabolic Diseases Networking Biomedical Research Centre (CIBERDEM), Institute of Health Carlos III (ISCIII), Av. Monforte de Lemos 3-5, 28029 Madrid, Spain

**Keywords:** primary hypercholesterolemia, cytokines, chemokines, leukocyte activation, platelet activation, endothelial dysfunction, systemic inflammation

## Abstract

Primary hypercholesterolemia (PH) is associated with a low grade systemic inflammation that is likely the main driver of premature atherosclerosis. Accordingly, we characterized the immune cell behaviour in PH and its potential consequences. Whole blood from 22 PH patients and 21 age-matched controls was analysed by flow cytometry to determine the percentage of leukocyte immunophenotypes, activation, and platelet-leukocyte aggregates. Plasma markers were determined by Enzyme-Linked ImmunoSorbent Assay (ELISA). The adhesion of platelet-leukocyte aggregates to tumor necrosis factor-α (TNFα)-stimulated arterial endothelium was investigated using the dynamic model of the parallel-plate flow chamber. PH patients presented greater percentage of Mon 3 monocytes, Th2 and Th17 lymphocytes, activated platelets, and leukocytes than controls. The higher percentages of circulating platelet-neutrophil, monocyte and lymphocyte aggregates in patients caused increased platelet-leukocyte adhesion to dysfunctional arterial endothelium. Circulating CXCL8, CCL2, CX_3_CL1, and IL-6 levels positively correlated with key lipid features of PH, whereas negative correlations were found for IL-4 and IL-10. We provide the first evidence that increased platelet and leukocyte activation leads to elevated platelet-leukocyte aggregates in PH and augmented arterial leukocyte adhesiveness, a key event in atherogenesis. Accordingly, modulation of immune system behavior might be a powerful target in the control of further cardiovascular disease in PH.

## 1. Introduction

Cardiovascular disease (CVD), predominantly coronary heart disease (CHD) and stroke, remains the main cause of death in most European countries [1], and atherosclerosis is the most common pathologic process for myocardial and cerebral ischemic disorders [2]. In recent years, it has become evident that systemic inflammation is the main driver of premature atherosclerosis and its complications, together with elevated plasma levels low density lipoprotein (LDL)-cholesterol [3].

Primary hypercholesterolemia (PH) is a lipid disorder characterized by elevated serum levels of cholesterol and low-density lipoprotein (LDL). This metabolic disorder is heterogeneous at the genetic level and includes both autosomal-dominant familial hypercholesterolemia (ADH), which has an approximate prevalence of 1:200–1:500, and the more frequent polygenic non-familial hypercholesterolemia [4]. In general, deleterious environmental factors such as a hypercholesterolemic diet and obesity are linked to the disease.

Previous studies indicate that low grade systemic inflammation is associated with PH, which might explain the higher incidence of CVD in these patients [4,5]. In this context, several studies have shown that different soluble inflammatory markers, including tumor necrosis factor-α (TNFα), interleukin-1 (IL-1), IL-6, interferon-γ (IFN-γ) and high sensitivity C reactive protein, are detected at higher levels in patients with hypercholesterolemia than in age-matched controls [5,6,7,8,9,10,11]. In addition, it is widely accepted that different chemotactic cytokines or chemokines are involved in the initial stage of the atherosclerotic lesion formation and more precisely in the recruitment of different leukocyte subsets [12]. In patients with hypercholesterolemia, increased circulating concentrations of monocyte chemoattractant protein-1 (MCP-1/CCL2), macrophage inflammatory protein-1α (MIP-1α/CCL3), MIP-1β (CCL4), IL-8/CXCL8 and IFN-γ-inducible protein10 (IP-10/CXCL10) have been also described [10,11]. By contrast, whereas the mRNA expression of regulated on activation, normal T cell expressed and secreted (RANTES/CCL5) was reported to be increased in mononuclear cells from children with familial hypercholesteromia, this chemokine mRNA up-regulation was not found in adults with the disease [13].

Despite these findings, little is known about the different cellular components of the systemic inflammatory response present in PH and their clinicopathological consequences. Given that a richer understanding of immune system behavior might open new horizons for CVD prognosis and treatment, in the present study we performed an exhaustive analysis of different cellular and soluble immune players in patients with PH and their potential consequences in arterial leukocyte adhesion, a crucial event in atherogenesis. The activation state of platelets and relevant leukocyte subsets have been explored as well as their interactions that lead to the formation of platelet-leukocyte aggregates. Additionally, the different circulating levels of cytokines and chemokines involved in the activation state of these cellular inflammatory components or in their recruitment have been quantified and correlations with key lipid components of the disease have been established. 

One of the earliest stages of atherogenesis is endothelial dysfunction, a proinflammatory and prothrombotic phenotype of the endothelium that leads to platelet activation and the adhesion and subsequent migration of T cells and leukocytes to the subendothelial space [14]. A correlation has been previously reported between inflammation and endothelial dysfunction in patients with PH [5]. Because very little is known about platelet-leukocyte-endothelium and leukocyte-endothelium interactions in PH, we also sought to evaluate the functional significance of the inflammatory status in PH using an *ex vivo* model of dysfunctional endothelium.

## 2. Materials and Methods

### 2.1. Cell culture

Human umbilical arterial endothelial cells (HUAEC) were isolated by collagenase treatment. Details are described in the Supplemental data.

### 2.2. Human Study Populations

A total of 43 subjects (22 PH patients and 21 age-matched control subjects without PH) were included in the present study. Patients and control volunteers were recruited at the Endocrinology Unit at University Clinic Hospital of Valencia, Spain.

Human studies were performed following the principles outlined in the Declaration of Helsinki and were approved by the Clinical Research Ethics Committee of the University Clinical Hospital of Valencia, Spain. All patients/controls signed an informed consent to participate in the study. Further details are described in the Supplemental data.

### 2.3. Flow Cytometry

Full details are described in the Supplemental data, including the gating strategy (Figures S1–S6 and Table S2).

### 2.4. Quantification of Soluble Metabolic and Inflammatory Markers

Heparinized whole blood from patients and controls was used to quantitatively measure different soluble metabolic and inflammatory markers by Enzyme-Linked ImmunoSorbent Assay (ELISA). Further details are described in the Supplemental data.

### 2.5. Leukocyte-Endothelial Cell Interactions under Flow Conditions

Whole blood, treated or not with EDTA (Panreac, Barcelona, Spain), 10 mM, 15min, 37 °C), was perfused across endothelial monolayers unstimulated or stimulated with TNFα (20 ng/mL, Sigma-Aldrich, Madrid, Spain), for 24 h. Details are described in the Supplemental data.

### 2.6. Immunofluorescence Studies

Details are described in the Supplemental data.

### 2.7. Statistical Analysis

All results were analyzed using GraphPad Prism software (GraphPad Software, Inc., La Jolla, CA, USA). Values are expressed as individual data points, percentages or mean ± standard error of the mean (SEM) when appropriate. For two-group comparisons, paired or unpaired Student´s t test was used in data that passed both normality (Kolmogorov*-*Smirnov) and equal variance (Levene) tests, as appropriate; otherwise, the non-parametric Mann Whitney *U* test was performed. For comparisons among multiple groups, one-way analysis of variance (ANOVA) followed by post hoc Bonferroni analysis was used in data that passed both normality and equal variance tests; otherwise, the non-parametric Kruskal-Wallis test followed by Dunn´s post hoc analysis was used. Data were considered statistically significant at *p* < 0.05.

## 3. Results

A total of 43 subjects (22 patients with PH and 21 age-matched control subjects without PH) were included in the present study. The demographic, clinical and laboratory characteristics of patients and controls are shown in Table 1. No statistically significant differences were found with regards to age, gender, body mass index (BMI) or waist circumference between the two groups (Table 1). By contrast, levels of total cholesterol (TC), LDL, triglycerides (TG) and apolipoprotein B (ApoB) were significantly higher in patients than in controls (Table 1). 

### 3.1. Platelet Activation Is Enhanced in Patients with PH 

**Figure 1:**
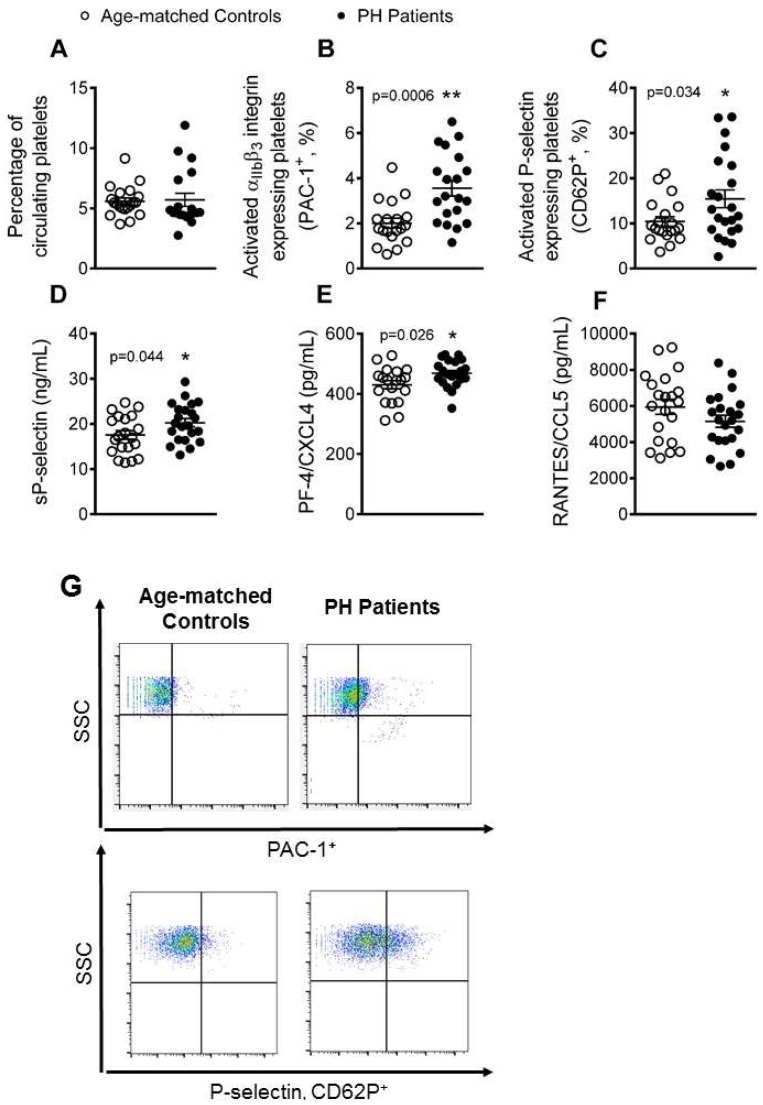
Platelet activation and related soluble markers are elevated in patients with PH. Flow cytometry analysis of platelets stained with conjugated antibodies against CD41 (**A**), CD41 and PAC-1 (**B** and **G**), and CD41 and P-selectin (**C** and **G**). Results are expressed as percentage of positive cells. Soluble P-selectin (sP-selectin, **D**), PF-4/CXCL4 (**E**) and RANTES/CCL5 (**F**) plasma levels (ng or pg/mL) were measured by ELISA. (*n* = 21 control subjects and *n* = 22 PH patients). Values are expressed as mean ± SEM. SSC: Side Scatter. * *p* < 0.05 or ** *p* < 0.01 relative to values in the control group.

We first determined the platelet activation state and levels of several mediators released upon their activation in blood samples from the two study groups using flow cytometry and ELISA. No significant differences in the number of circulating platelets were found between controls and patients (Figure 1A). By contrast, the percentage of platelets expressing PAC-1 and P-selectin (CD62P) was significantly higher in patients than in controls (Figure 1B, C and G), indicating their activation. Since P-selectin translocates to the cell surface upon cell activation, where it can be cleaved and released into the circulation as soluble P-selectin (sP-selectin), we also determined its circulating levels in plasma, finding that levels were significantly higher in the PH group than in the control group (Figure 1D). Similarly, circulating plasma levels of platelet factor-4 (PF-4/CXCL4), a platelet chemokine released upon platelet activation, were significantly higher in PH patients than in controls (Figure 1E). No differences, however, were encountered between PH patients and control subjects for the levels of circulating RANTES (regulated on activation, normal T cell expressed and secreted)//CCL5, a chemokine released by platelets and other immune cells when activated (Figure 1F).

### 3.2. The Percentage of Platelet-Neutrophil Aggregates, Activated Neutrophils, and Circulating Levels of IL-8, Are Elevated in Patients with PH

**Figure 2:**
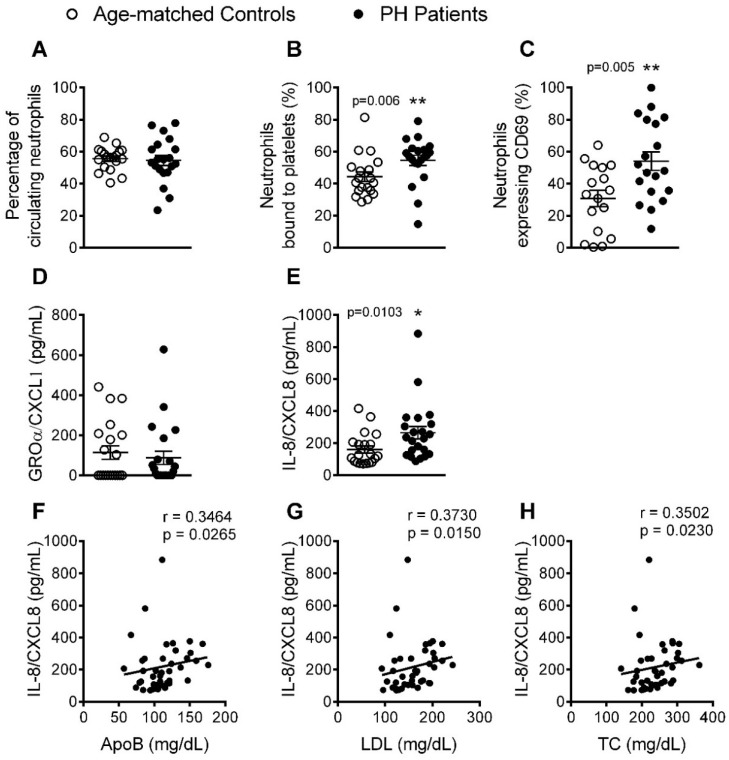
The percentage of platelet-neutrophil aggregates and activated neutrophils, and IL-8 circulating levels, are higher in patients with PH. Flow cytometry analysis of heparinized whole blood co-stained with specific markers for platelets and neutrophils (**A** and **B**). Neutrophils were also stained for CD69 (**C**). Results are expressed as percentage of positive cells. GROα/CXCL1 (**D**) and IL-8/CXCL8 (**E**) plasma levels (pg/mL) were measured by ELISA (*n* = 21 control subjects and *n* = 22 PH patients). Values are expressed as mean ± SEM. * *p* < 0.05 or ** *p* < 0.01 relative to values in the control group. Correlations between circulating IL-8 and ApoB (**F**), LDL (**G**) and TC (**H**) plasma levels.

We next evaluated several parameters related to the activation of different leukocyte subsets. No significant differences were found in the percentage of circulating neutrophils in heparinized blood between the two groups (Figure 2A); however, the percentage of platelet-neutrophil aggregates and activated neutrophils (CD69^+^) was significantly higher in patients than in controls (Figure 2B,C). As some chemokines, such as growth-regulated oncogene-α (GROα/CXCL1) and IL-8 (CXCL8), can induce activation and chemotaxis of human neutrophils, we quantified their levels in plasma. Whereas no differences in the levels of CXCL1 were detected between the two groups (Figure 2D), plasma levels of IL-8 were significantly elevated in PH patients (Figure 2E). Of note, we found a significant association between the circulating levels of IL-8 and three clinical features of PH in patients: ApoB, LDL and TC (Figure 2F–H).

### 3.3. Circulating Mon 3 Monocytes, Platelet-Mon 1 and 3 Aggregates, Activated Mon 1 and 2 Monocytes, and Plasma Levels of CCL2 and CX_3_CL1, Are All Elevated in Patients with PH 

**Figure 3:**
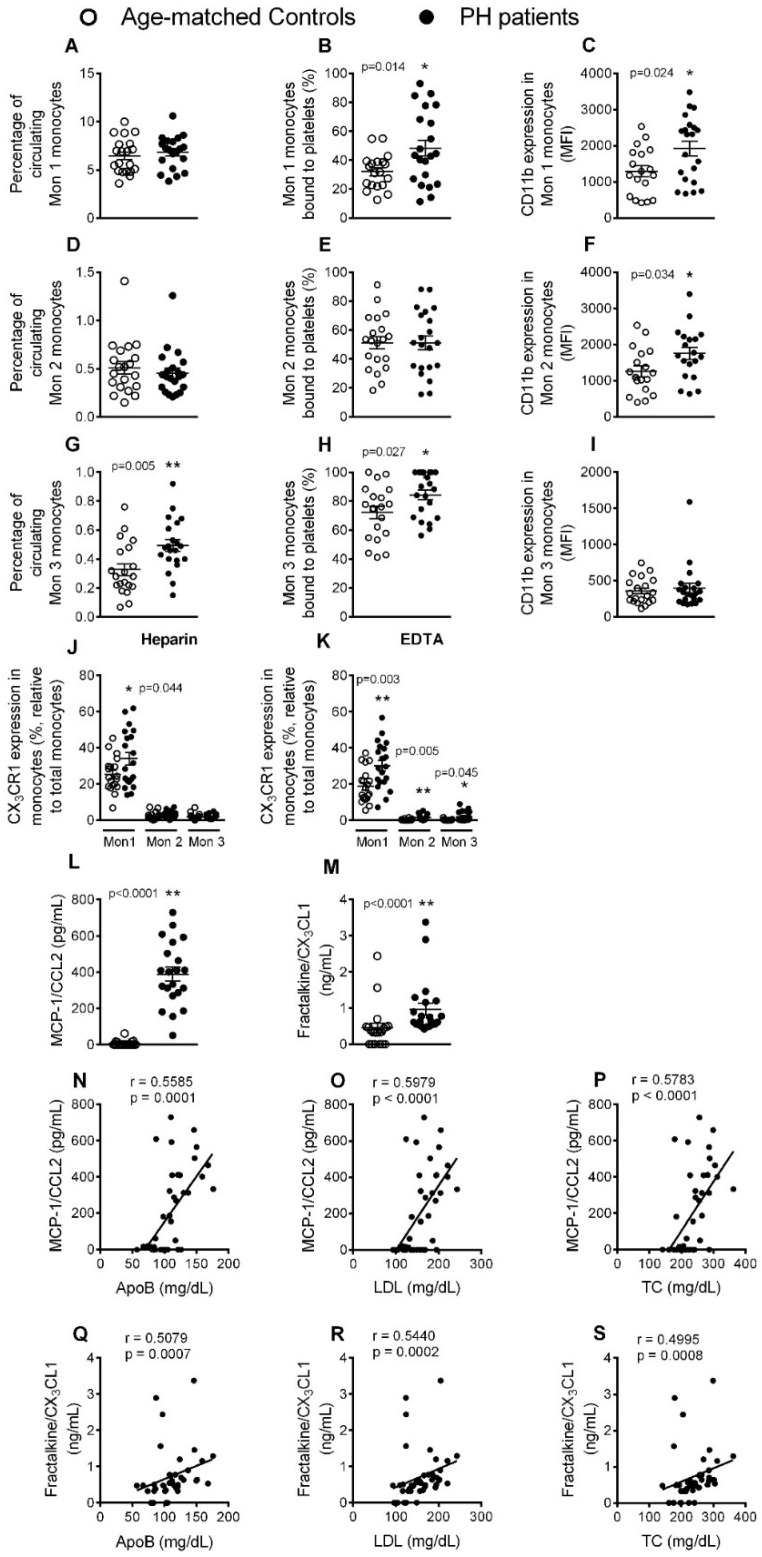
The percentage of circulating Mon 3 monocytes, platelet-Mon 1 and 3 aggregates, activated Mon 1 and 2 monocytes, and plasma levels of CCL2 and CX_3_CL1, are elevated in patients with PH. Flow cytometry analysis of heparinized whole blood co-stained with specific markers for platelets and Mon 1, 2 and 3 monocytes (**A**, **B**, **D**, **E**, **G**, **H**), CD11b integrin (**C**, **F** and **I**), and also for CX_3_CR1 in heparinized and EDTA-treated whole blood (**J** and **K**). Results are expressed as percentage of positive cells or mean fluorescence intensity (MFI). MCP-1/CCL2 (**L**) and fractalkine/CX_3_CL1 (**M**) plasma levels (pg or ng/mL) were measured by ELISA (*n* = 21 control subjects and *n* = 22 PH patients). Values are expressed as mean ± SEM. * *p* < 0.05 or ** *p* < 0.01 relative to values in the control group. Correlations between circulating MCP-1/CCL2 or fractalkine/CX_3_CL1 and ApoB (**N** and **Q**), LDL (**O** and **R**) and TC (**P** and **S**) plasma levels.

Three monocyte subpopulations have been described in peripheral blood based on their differential expression of the cell surface markers CD14, CD16 and CCR2 (Table S1, Supplemental data). Whereas the percentage of circulating type 1 (Mon 1) and 2 (Mon 2) monocytes in blood was not different between patients and controls, as determined by flow cytometry, we found a significantly higher percentage of circulating type 3 (Mon 3) monocytes in the former group (Figure 3A,D,G). When we analyzed platelet-monocyte aggregates, those established between platelets and Mon 1 and 3 monocytes were significantly elevated in patients with PH (Figure 3B,E,H). Moreover, the expression of CD11b integrin in Mon 1 and 2 monocytes, but not in Mon 3 monocytes, was significantly higher in patients than in controls, indicating their activation (Figure 3C,F,I). Analysis of the fractalkine/CX_3_CL1 receptor (CX_3_CR1) on the different monocyte subtypes from heparinized whole blood revealed that the percentage of Mon 1 monocytes expressing this receptor was significantly higher in patients than in controls (Figure 3J). After dissociating platelets with EDTA, we found that the percentage of all monocyte subtypes positive for CX_3_CR1 was significantly higher in patients than in controls, with Mon 1 monocytes showing the highest percentage of CX_3_CR1 expression (Figure 3K). In addition, the circulating levels of MCP-1/CCL2 and soluble fractalkine/CX_3_CL1, ligands of CCR2 and CX_3_CR1 receptors respectively and involved in mononuclear cell recruitment, were significantly higher in patients (Figure 3L,M), and a positive correlation was found between the circulating concentration of both chemokines and ApoB, LDL and TC levels in patients (Figure 3N–S).

### 3.4. Circulating CD4^+^ Lymphocytes, Platelet-Lymphocyte (CD4^+^ and CD8^+^) Aggregates and Lymphocyte (CD4^+^ and CD8^+^) Activation Are Significantly increased in Patients with PH 

**Figure 4:**
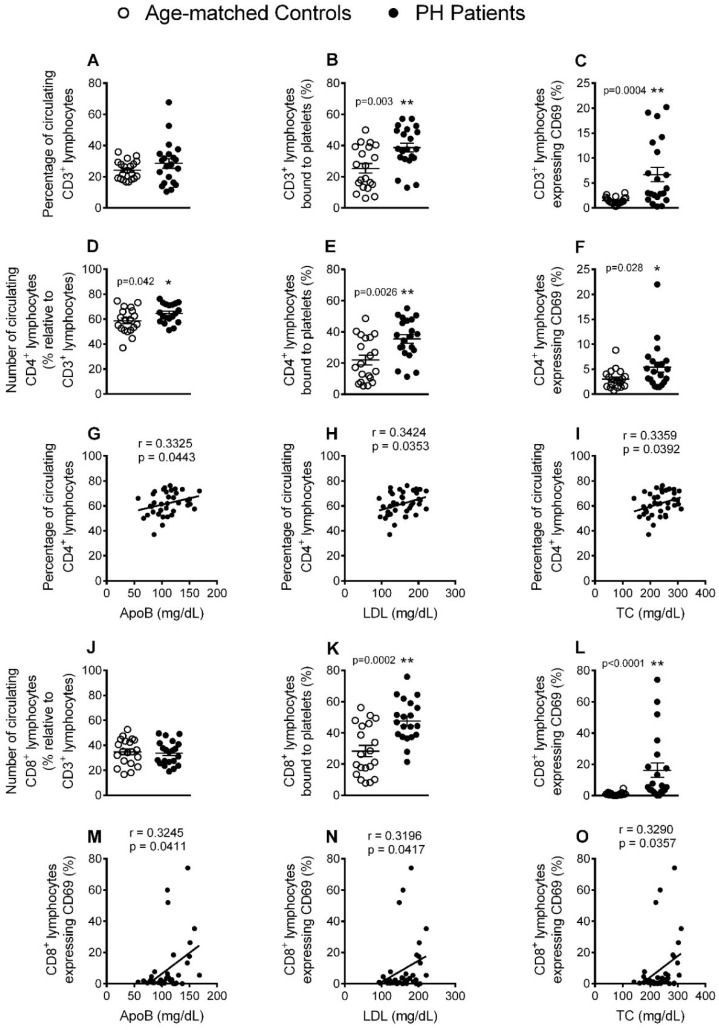
The percentage of circulating CD4^+^ lymphocytes, platelet-lymphocyte (CD4^+^ and CD8^+^) aggregates, and lymphocyte (CD4^+^ and CD8^+^) activation, are significantly elevated in patients with PH. Heparinized whole blood was co-stained with specific markers for platelets and CD3^+^, CD4^+^ and CD8^+^ lymphocytes (**A**, **B**, **D**, **E**, **J** and **K**) as well as for CD69 (**C**, **F** and **L**). Results are expressed as the percentage of positive cells (*n* = 21 control subjects and *n* = 22 PH patients). Values are expressed as mean ± SEM. * *p* < 0.05 or ** *p* < 0.01 relative to values in the control group. Correlations between circulating CD4^+^ cells and activated CD8^+^ lymphocytes and ApoB (**G** and **M**), LDL (**H** and **N**) and TC (**I** and **O**) plasma levels.

Mature T cells express the general marker CD3, and also express either CD4 or CD8 depending on the type of T cell. Whereas no differences were found in circulating numbers of CD3^+^ and CD8^+^ lymphocytes between patients and controls, the number of CD4^+^ lymphocytes was higher the former group (Figure 4A,D,J) and positively correlated with ApoB, LDL and TC levels (Figure 4G–I). Moreover, the percentage of CD3^+^, CD4^+^ and CD8^+^ lymphocytes bound to platelets, and also their activation state, (CD69^+^) was greater in patients than in control subjects (Figure 4B,C,E,F,K,L). Interestingly, a positive correlation was found between the percentage of CD69^+^CD8^+^ cells and the lipid profile (ApoB, LDL and TC levels) (Figure 4M–O).

**Figure 5:**
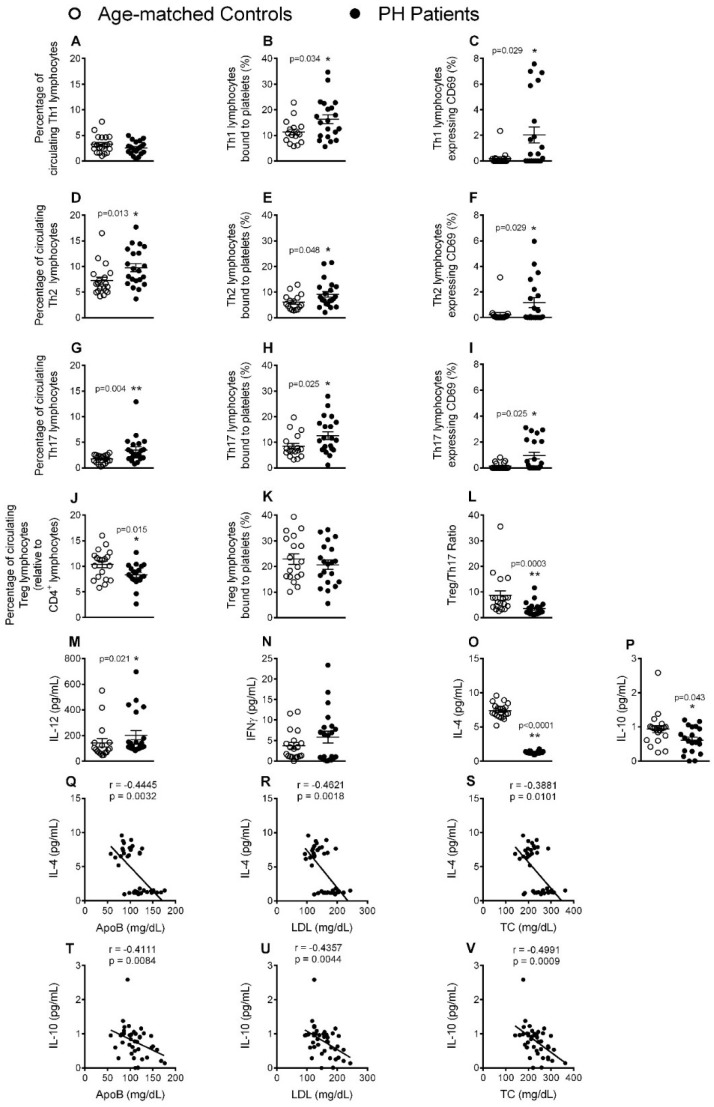
The percentage of circulating Th2 and Th17 lymphocytes, platelet-lymphocyte aggregates, lymphocyte activation and IL-12 circulating levels, are significantly increased in patients with PH, whereas the percentage of circulating Treg cells, Treg/Th17 ratio, and IL-4 and IL-10 plasma levels, are decreased. Heparinized whole blood was co-stained with specific markers for platelets and Th1, Th2, Th17 and Treg lymphocytes (**A**, **B**, **D**, **E**, **G**, **H**, **J** and **K**) as well as for CD69 (**C**, **F** and **I**). Treg/Th17 ratio was also determined (**L**). Results are expressed as percentage of positive cells. IL-12 (**M**), IFNγ (**N**), IL-4 (**O**) and IL-10 (**P**) plasma levels (pg/mL) were measured by ELISA (*n* = 21 control subjects and *n* = 22 PH patients). Values are expressed as mean ± SEM. * *p* < 0.05 or ** *p* < 0.01 relative to values in the control group. Correlations between circulating IL-4 or IL-10 and ApoB (**Q** and **T**), LDL (**R** and **U**) and TC (**S** and **V**) plasma levels.

Closer inspection of the different CD4^+^ T lymphocyte subtypes revealed an increased number of circulating Th2 and Th17, but not Th1, lymphocytes in patients (Figure 5A,D,G). Furthermore, we found that the percentage of circulating platelet-Th lymphocyte aggregates of all three subtypes, as well as their activation state (CD69^+^), was higher in patients than in controls (Figure 5B,C,E,F,H,I). In contrast to CD4^+^ cells, the percentage of circulating Treg lymphocytes (Figure 5J), as well as the Treg/Th17 ratio (Figure 5L), was significantly lower in patients. However, no differences were found between patients and controls in the percentage of circulating Treg lymphocyte-platelet aggregates (Figure 5K). Of note, whereas the circulating levels of IL-12, a cytokine involved in the differentiation of naïve T cells to Th1 cells, were significantly elevated in patients, plasma levels of IFNγ, a cytokine released by Th1 lymphocytes, were not different from those of control subjects (Figure 5M,N). By contrast, levels of the anti-inflammatory cytokines IL-4 and IL-10, which are mainly produced by Th2 and Treg lymphocytes, respectively, were significantly lower in the circulation of PH patients (Figure 5O,P). Indeed, an inverse correlation was found between IL-4 and IL-10 and the lipid profile associated with PH (Figure 5Q–V). 

### 3.5. Circulating Levels of Pro-Inflammatory Cytokines but not Adipokines Are Increased in PH Patients

**Figure 6:**
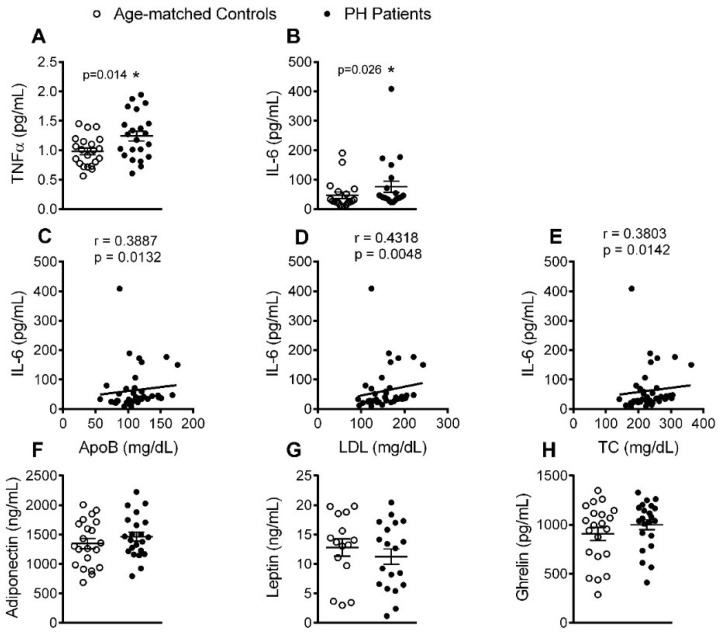
Increased circulating levels of pro-inflammatory cytokines but not adipokines in patients with PH. TNFα (**A**), IL-6 (**B**), adiponectin (**F**), leptin (**G**) and ghrelin (**H**) plasma levels (pg or ng/mL) were measured by ELISA (*n* = 21 control subjects and *n* = 22 PH patients). Values are expressed as mean ± SEM. * *p* < 0.05 or ** *p* < 0.01 relative to values in the control group. Correlations between circulating IL-6 and ApoB (**C**), LDL (**D**) and TC (**E**) plasma levels.

Th17 cells produce TNFα and IL-6 [15], and an increase in the plasma levels of these pro-inflammatory cytokines has been reported in patients with PH [6,7,9]. We noted similar findings in our patient cohort (Figure 6A,B); moreover, a positive association was found between IL-6 plasma levels and the levels of circulating ApoB, LDL and TC (Figure 6C–E). By contrast, no differences were found for the circulating levels of adiponectin, leptin or ghrelin between patients and controls (Figure 6F–H). 

### 3.6. Circulating Platelet-Leukocytes and Leukocytes from PH Patients Have Increased Adhesiveness to TNFα-Stimulated HUAEC 

Endothelial dysfunction is one of the earliest stages of atherogenesis, and leads to the adhesion and the subsequent migration of leukocytes [14]. Because TNFα is a central cytokine/adipokine in hypercholesterolemia [9,11], we next explored the functional consequences of the elevated levels of TNFα in patients with PH. We first examined the adhesion of platelet-leukocyte aggregates and leukocytes alone to unstimulated or TNFα-stimulated arterial endothelial cells (HUAEC) under dynamic flow conditions. To do this, experiments were carried out with heparinized or EDTA-treated blood, which promotes the dissociation of platelets from leukocytes.

**Figure 7:**
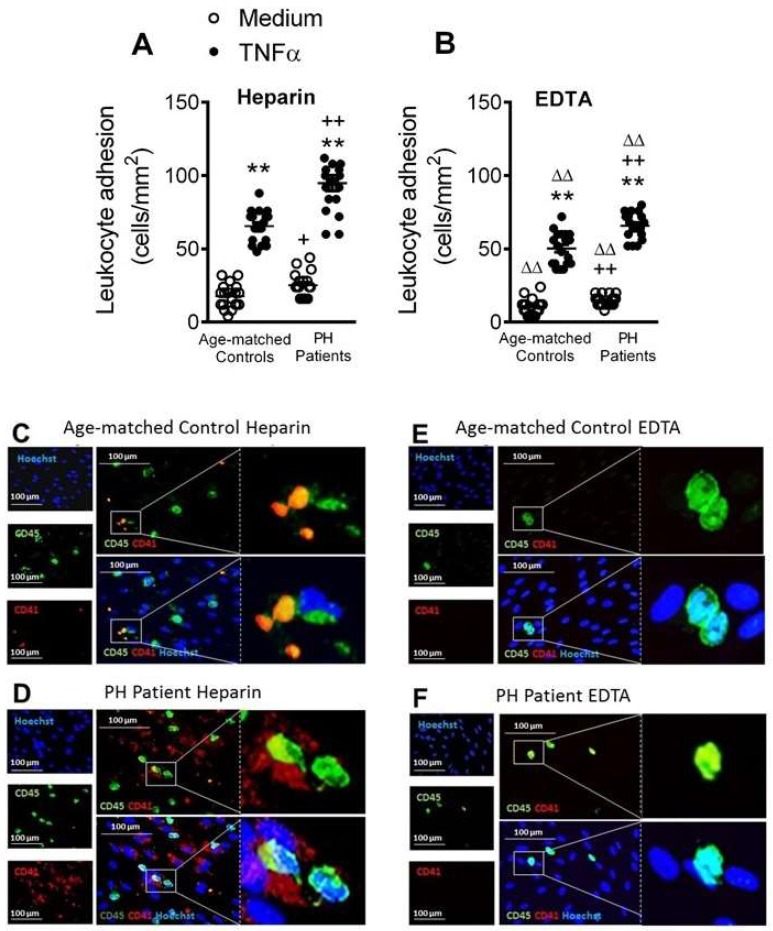
Circulating platelet-leukocyte aggregates and leukocytes from PH patients show increased adhesiveness to TNFα–stimulated HUAEC. HUAEC were stimulated or not with TNFα (20 ng/mL) for 24 h. Subsequently, whole blood from patients and controls, incubated without (**A**) or with EDTA (**B**), was perfused across endothelial monolayers for 7 min at 0.5 dyn/cm^2^ and leukocyte adhesion quantified (cells/mm^2^). Values are expressed as mean ± SEM (*n* = 21 control subjects and *n* = 22 PH patients). ** *p* < 0.01 relative to values in the medium group; +*p* < 0.05 or ++*p* < 0.01 relative to respective values in the control group; ∆∆*p* < 0.01 relative to respective values in the heparin group. Immunofluorescence analysis showing adherent platelet–leukocyte cell complexes to TNFα-stimulated HUAEC (**C**–**F**). Heparinized blood from age-matched controls and patients with PH was incubated without or with EDTA. After the flow chamber assay, cells were fixed with 4% paraformaldehyde and blocked in phosphate-buffered saline (PBS) containing 1% bovine serum albumin (BSA). Then, cells were incubated for 2 h with an Alexa 488-conjugated antibody against human CD45 (1:50 dilution, green) and an allophycocyanin (APC)-conjugated antibody against human CD41 (1:50 dilution, red). Nuclei of endothelial cells and leukocytes were stained with Hoechst (blue). Images were captured with a Zeis Axio Observer A1 fluorescence microscope.

When heparinized, diluted whole blood from patients and controls was perfused across unstimulated HUAEC, leukocyte adhesiveness was significantly greater in the PH group (Figure 7A). After exposure of HUAEC to TNFα for 24 h, leukocyte adhesiveness increased in both groups and remained significantly greater in the PH group (Figure 7A). Importantly, when platelets were disaggregated from leukocytes with EDTA, leukocyte adhesion was still significantly greater in the PH group than the control group (Figure 7B), despite the significant decrease in the number of adhered leukocytes to stimulated HUAEC after platelet disaggregation (Figure 7A,B). In agreement with these observations, immunofluorescence studies revealed enhanced adherent platelet–leukocyte complexes to TNFα-stimulated endothelial cells from PH patients compared with age-matched controls (Figure 7C,D). Furthermore, when platelets were disaggregated from leukocytes with EDTA, leukocyte adhesion to TNFα-stimulated HUAEC was notably diminished but this parameter was markedly greater in the PH group than the control group (Figure 7E,F).

## 4. Discussion

PH is associated with risk of developing arteriosclerosis and the likelihood of future serious ischemic events. Previous studies have provided evidence of low systemic inflammation in patients with hypercholesterolemia [6,7,9,10,11,16]. Here, we carried out a detailed characterization of different immune players and soluble inflammatory markers in PH and correlated these data with the circulating levels of key lipid components. The enhanced inflammatory status of PH reported herein has functional consequences, as illustrated for circulating platelet-bound leukocytes, which have increased adhesiveness to dysfunctional arterial endothelium, a prominent feature of the atherogenic process.

Platelet activation is known to be associated with atherogenesis and cardiovascular morbidity [17]. Indeed, upon their activation, platelets express specific cell adhesion molecules such as P-selectin, and release several inflammatory chemokines including PF-4/CXCL4 or RANTES/CCL5 [17]. We show that patients with PH present a pro-thrombotic state characterized by increased platelet activation (P-selectin^+^ and PAC-1^+^ platelets). While hypercholesterolemia has been previously associated with platelet activation [5,16], we found that patients have both increased circulating levels of sP-selectin and PF-4/CXCL4, which are involved in multiple atherogenic processes. Indeed, different platelet surface molecules such as GPIIb/IIIa (recognized by PAC-1) or P-selectin are critically involved in the interaction of platelets to endothelial cells and leukocytes [17], all of which are central for atherosclerotic lesion formation. 

To gain insight into the immune state of the hypercholesterolemic environment of PH, we examined different leukocyte subtypes. An increase in leukocyte activation *in vitro* has been reported in subjects at high cardiovascular risk (hyperlipidemia) [18]. In our study, whereas no differences in the percentage of circulating neutrophils were detected between patients and controls, a clear increase in the percentage of activated cells (CD69^+^) was observed, suggesting the existence of a proatherogenic state. This is consistent with our finding of increased circulating levels of CXCL8, which is involved in neutrophil activation, in the PH group, as has been reported previously, albeit in patients with FH [10]. Also, the plasma concentrations of this chemokine positively correlated with the circulating levels of key lipids in PH, ApoB, LDL and TC. Overall, these results indicate that IL-8 might have utility as biomarker of atherosclerotic risk in PH.

Human monocytes are a heterogeneous cell population that are commonly classified into three subtypes: classical CD14^+^CD16^–^CCR2^+^ (Mon 1), intermediate CD14^+^CD16^+^CCR2^+^ (Mon 2), and nonclassical CD14^+^CD16^+^CCR2^-^ (Mon 3) [19]. There is evidence to support that adults with FH have a pro-inflammatory imbalance in circulating monocyte subpopulations (Mon 1) [20], although another study indicated that the levels of Mon 2 and/or Mon 3 subtypes were increased in hyperlipidemia and associated with atherosclerosis development [21]. We found that only the percentage of the nonclassical/Mon 3 subtype was increased in patients over controls, and this positively correlated with the circulating levels of apoB, LDL and TC (Figure S7, Supplemental data). By contrast, Mon 1 and Mon 2 subtypes, both of which express the CCR2 receptor, were significantly activated in patients. We also show for the first time an increase in the percentage of fractalkine/CX_3_CL1 receptor (CX_3_CR1) expression on Mon 1 monocytes in heparinized whole blood, and on all monocyte subsets when platelets were dissociated. In line with these observations, studies of atherosclerosis in mice suggest that both inflammatory (similar to human Mon 1) and patrolling (similar to human Mon 3) monocytes are involved in disease progression [22]. In humans, different studies have noted increases in circulating CD16^+^ monocytes in cardiovascular disease [22], which are possibly linked to disease outcome [23]. There is also evidence to support that mobilized classical monocytes from the bone marrow mature into nonclassical monocytes through an intermediate subset. How these different monocyte subtypes correlate with disease pathogenesis and clinical outcomes in PH is, however, unknown. Nevertheless, it is likely that those subtypes expressing both CCR2 and CX_3_CR1 are more prone to migrate from the circulation into arterial walls through the interaction with their cognate ligands CCL2 and CX_3_CL1, the circulating levels of which were significantly elevated in patients and correlated positively with plasma ApoB, LDL and TC content. 

To the best of our knowledge, the associations we found between T lymphocytes and PH have not previously been reported. Four findings are worthy of mention. First, the percentage of circulating CD4^+^ cells was significantly higher in patients with PH than controls, and directly correlated with levels of ApoB, LDL and TC. Of note, this increase was likely due to the increased numbers of circulating Th2 and Th17 cells. Second, most of the T cell subpopulations in patients displayed an activated state and positive correlations were found between the percentage of CD8^+^ CD69^+^ cells and key lipid features of the disease. Third, the percentage of circulating Treg cells and the Treg/Th17 ratio was decreased in PH patients. Finally, whereas IL-12, TNFα and IL-6 plasma levels were increased in patients, levels of the anti-inflammatory cytokines IL-4 or IL-10 were decreased and inversely correlated with the levels of ApoB, LDL and TC. These observations, overall, link the cellular and molecular inflammatory profile to a possible pro-atherogenic enviroment. Along this line, it is well known that both CD4^+^ and CD8^+^ T cells are involved in atherosclerosis development [24]. While the role of Th2 cells in atherogenesis remains debated [24], it has recently been shown that patients with coronary artery atherosclerosis had an impaired Treg/Th17 ratio together with reduced serum levels of IL-10 [25]. Th17 cells repress the function of Treg cells, contributing to an inflammatory milieu. Moreover, whereas Th17 cells can produce the inflammatory cytokines TNFα and IL-6 [15], Treg cells generate and release the anti-inflammatory cytokine IL-10. It is therefore tempting to speculate that there is a conversion of Treg cells into Th17 cells in PH. Finally, although IL-4 is a classic Th2 cytokine, the decreased levels found in patients suggest an alternative cellular origin of this cytokine. Indeed, potential sources of IL-4 are double-positive CD4/CD8 lymphocytes, basophils or natural killer T cells [26,27] whose circulating levels may be decreased in this pathology, although this requires further investigation. 

We used a dynamic flow chamber model to explore the functional consequences of platelet-leukocyte-endothelium (heparin) or leukocyte-endothelium (EDTA) interactions, finding that adhesion of platelet-leukocyte aggregates to HUAEC, stimulated or not with TNFα, was significantly higher in the patient group. The increased adhesion to functional (nonstimulated) endothelium was likely due to neutrophil and monocyte activation and consequent over-expression of CD11b/CD18 integrin, which interacts with the constitutively expressed intercellular cell adhesion molecule-1 in endothelium. Furthermore, platelets seem to be critical for leukocyte adhesion to dysfunctional (stimulated) arterial endothelium, as leukocyte-endothelium interactions were significantly impaired when platelets were dissociated with EDTA. It is widely accepted that activated platelets can mediate the endothelial adhesion of circulating leukocytes, a characteristic feature of the dysfunctional endothelium [14,28,29,30,31]. We also found a significant enhancement in the percentage of platelet-leukocyte aggregates, which were established with almost all the leukocyte subsets investigated in a background of PH. The increased number of these aggregates have been detected in the peripheral circulation of patients with unstable angina or other coronary diseases, and they have been considered a predictive factor of acute myocardial infarction [32]. This platelet-leukocyte interaction is in all probability due to the interaction of platelet P-selectin with its ligand, P-selectin glycoprotein ligand-1, present on leukocyte surfaces, which in turns facilitates the interaction between these aggregates and dysfunctional endothelium, a key event in arteries prone to arteriosclerotic lesion development [14]. 

In conclusion, we report that the low grade systemic inflammation associated with PH is accompanied by a pro-thrombotic state with heightened platelet activation and associated circulating soluble markers. This platelet activation state in PH, together with the activation of different leukocyte subsets, results in the formation of platelet-leukocyte aggregates and their adhesion to dysfunctional arterial endothelium, suggesting a potential link between systemic inflammation and CVD development in this metabolic disorder. Finally, the positive correlations between key lipid features of PH and different circulating inflammatory mediators (IL-8, MCP-1, fractalkine or IL-6) and the negative correlations between these lipids and anti-inflammatory cytokines (IL-4 and IL-10) might be used as potential markers of CVD. Overall, the modulation of the cellular and molecular inflammatory components in PH, as well as the lipid profile, might be crucial to prevent further cardiovascular complications.

## Figures and Tables

**Table 1:** Demographic and clinical features of patients and age-matched controls.

	Control Volunteers (*n* = 21)	PH Subjects (*n* = 22)	*p* Value
**Age (years)**	48.8 ± 2.7	49 ± 3.1	0.95
**Gender M/F (%)**	5/16 (23.8/76.2)	4/18 (18.2/81.8)	0.72
**BMI (kg/m^2^)**	25.4 ± 0.7	25.7 ± 0.9	0.83
**Waist circumference (cm)**	85.3 ± 1.9	85.7 ± 2.2	0.90
**SBP (mmHg)**	115.9 ± 2.0	124.7 ± 3.6 *	**<0.05**
**DBP (mmHg)**	71.6 ± 1.8	78.5 ± 2.6 *	**<0.05**
**Glucose (mg/dL)**	86.7 ± 1.5	88.1 ± 1.9	0.57
**TC levels (mg/dL)**	206.1 ± 6.8	264.6 ± 8.9 **	**<0.01**
**LDL levels (mg/dL)**	130.6 ± 5.4	182.8 ± 6.2 **	**<0.01**
**TG (mg/dL)**	80.9 ± 7.3	109.7 ± 8.5 **	**<0.01**
**HDL levels (mg/dL)**	65.9 ± 2.5	63.4 ± 2.9	0.51
**ApoB (mg/dL)**	92.5 ± 4.1	127.4 ± 5.0 **	**<0.01**
**GOT (U/L)**	21.7 ± 0.9	22.8 ± 1.1	0.42
**GPT (U/L)**	18.3 ± 1.8	18.5 ± 1.1	0.90
**Creatinine (mg/dL)**	0.7 ± 0.0	0.7 ± 0.0	0.48
**IgG (mg/dL)**	966.7 ± 41.1	968.5 ± 34.4	0.97
**Igm (mg/dL)**	100.4 ± 7.6	125.8 ± 14.1	0.14
**IgE total (IU/L)**	42.6 ± 12.0	50.4 ± 16.9	0.71

BMI, body mass index; SBP, systolic blood pressure; DBP, diastolic blood pressure; TC, total cholesterol; LDL, low density lipoprotein; TG, triglycerides; HDL, high density lipoprotein; ApoB, apolipoprotein B; GOT, glutamic oxalacetic transaminase; GPT, glutamate-pyruvate transaminase; Ig, immunoglobulin. Data are presented as mean ± SEM. * *p* < 0.05 or ** *p* < 0.01 relative to values in the control group.

## Supplementary Materials

The following are available online at http://www.mdpi.com/2077-0383/8/1/18/s1, Figure S1. Gating strategy for human platelets in whole blood according to morphological properties and CD41 detection by flow cytometry, Figure S2. Gating strategy for human neutrophils in whole blood according to morphological properties and CD16 expression by flow cytometry, Figure S3. Gating strategy for human monocyte detection in whole blood by flow cytometry, Figure S4. Gating strategy for human T lymphocyte detection in whole blood by flow cytometry, Figure S5. Gating strategy for human T helper lymphocyte detection in whole blood by flow cytometry, Figure S6. Gating strategy for human regulatory T lymphocyte (Treg) detection in whole blood by flow cytometry, Figure S7. Positive correlation between the percentage of nonclassical/Mon 3 monocytes and the circulating levels of ApoB (A), LDL (B) and TC (C). Table S1. Differential markers of monocyte subpopulations, Table S2. Differential markers for detection of Th lymphocyte subpopulations.
