# Supplementary material for: Novel Immune Features of the Systemic Inflammation Associated with Primary Hypercholesterolemia: Changes in Cytokine/Chemokine Profile, Increased Platelet and Leukocyte Activation
Source: J Clin Med. 2018 Dec 22;8(1):18. doi: 10.3390/jcm8010018 (PMC6352074; doi:10.3390/jcm8010018)
Supplement: Supplementary file 1 [file jcm-08-00018-s001.pdf]

Journal of
Clinical Medicine

MDPI

Article

Novel Immune Features of the Systemic Inflammation Associated with Primary Hypercholesterolemia: Changes in Cytokine/Chemokine Profile, Increased Platelet and Leukocyte Activation

Aida Collado [1,3,†], Patrice Marques [1,3,†], Elena Domingo [1,†], Eva Perello [3,4], Herminia González-Navarro [3,4], Sergio Martinez-Hervás [2,3,4], José T. Real [2,3,4], Laura Piqueras [1,3], Juan F. Ascaso [2,3,4,*] and Maria-Jesus Sanz [1,3,4,*]

[1] Department of Pharmacology, Faculty of Medicine and Odontology, University of Valencia, Av. Blasco Ibáñez 15, 46010, Valencia, Spain; aida.collado@uv.es (A.C.); patricegmarques@gmail.com (P.M.); eledo2@hotmail.com (E.D.); laura.piqueras@uv.es (L.P.)
[2] Department of Medicine, Faculty of Medicine and Odontology, University of Valencia, Av. Blasco Ibáñez 15, 46010, Valencia, Spain; Sergio.Martinez@uv.es (S.M.-H.); Jose.T.Real@uv.es (J.T.R.)
[3] Institute of Health Research INCLIVA, University Clinic Hospital of Valencia, Av. Menéndez Pelayo 4, 46010, Valencia, Spain; evapc89@hotmail.com (E.P.); Herminia.Gonzalez@uv.es (H.G.-N.)
[4] CIBERDEM-Spanish Biomedical Research Centre in Diabetes and Associated Metabolic Disorders, ISCIII, Av. Monforte de Lemos 3-5, 28029, Madrid, Spain

Materials and methods

Human in vitro and ex vivo studies

Cell culture

Human umbilical arterial endothelial cells (HUAEC) were isolated by collagenase treatment [1] and maintained in human endothelial cell specific medium (EBM-2, Lonza, Barcelona, Spain), supplemented with endothelial growth media (EGM-2, Lonza) and containing 10% fetal bovine serum (FBS, Biowest, Nuaillé, France). Cells were grown to confluence up to passage 1 to preserve endothelial features. Prior to every experiment, cells were incubated for 24 h in medium containing 2% FBS.

Human study populations

The studies were performed following the principles outlined in the Declaration of Helsinki for the use of human subjects. The study protocol was approved by the Clinical Research Ethics Committee of the University Clinic Hospital of Valencia, Spain. All subjects signed an informed consent to participate in the study.

A total of 43 subjects (22 patients with primary hypercholeterolemia (PH) and 21 age-matched control subjects without PH) were included in the present study. Patients and control volunteers were recruited by the Endocrinology Unit of the University Clinic Hospital of Valencia, Spain. Diagnostic criteria for PH were the following: concentration of total plasma cholesterol (TC) >260 mg/dL and/or LDL >160 mg/dL and triglycerides (TG) <150 mg/dL. The inclusion criteria for the control group were the following: concentration of TC <200 mg/dL, TG <150 mg/dL and apoB <120 mg/dL; fasting plasma glucose <100 mg/dL and absence of personal or family history of dyslipidemia, cardiovascular disease or diabetes. Genetic testing for *APOE* was performed in both groups and only those subjects who presented the *E3/E3* genotype participated.

Exclusion criteria were the following: clinical manifestations of CHD, diabetes, hypertension, smoking, consumption of >30 g alcohol/day, participation in intense physical fitness or weight-loss programs, body-weight fluctuation >10% in the previous three months, other chronic diseases, other secondary hyperlipemias, renal or hepatic insufficiency and hypothyroidism, infection or inflammatory disease (including personal history of asthma or allergy) in the six weeks prior to the study, and the use of drugs capable of modifying the lipid profile or inflammation that cannot be withdrawn 6 weeks before starting the study.

Study design

A complete medical history, anthropometric and blood pressure measurements and a clinical chemistry analysis was performed to confirm eligibility. In the 6 weeks prior the study, any drug that could modify inflammation in patients was discontinued and patients were instructed to follow a cholesterol-lowering National Cholesterol Education Program-1 diet during this period. Control subjects maintained their usual dietary habits. The day before testing, all subjects abstained from alcohol intake and physical activity. The study started at 8:30 am, after a 12-hour overnight fast. Blood samples were obtained after resting for 30 min. Samples were drawn into either heparinized, ethylenediaminetetraacetic acid (EDTA) or sodium citrate tubes for analytical determinations, which included the following: blood cell differential counts, complete biochemistry with glycemic and lipid profile, and renal function through determination of creatinine levels. Demographic and clinical features of patients and age-matched controls are shown in Table 1.

Flow cytometry

Blood samples for flow cytometry studies were collected in BD Vacutainer® blood collection tubes containing 3.2% sodium citrate, or in BD Vacutainer® PST™ II tubes with lithium/heparin (17 IU/mL) as anticoagulant agents (both from BD Biosciences, San Jose, CA). All samples were run in a FACSVerse™ flow cytometer (BD Biosciences) and all flow cytometry data were analysed with FlowJo® v10.0.7 software (FlowJo LLC, Ashland, OR).

To determine platelet activation, PAC-1$^+$ platelets (detecting activated integrin $\alpha_{IIb}\beta_3$/GPIIb/IIIa) and the expression of P-selectin (CD62P) were measured in platelets by flow cytometry. Citrated blood samples (6.25 μL) were diluted 1:10 in glucose buffer (1 mg/mL glucose in PBS containing 0.35% BSA; Sigma-Aldrich, Madrid, Spain). Samples were incubated in the dark for 30 min with a 5-carboxyfluorescein (CF)-Blue™-conjugated monoclonal antibody (mAb) against human CD41 (1.25 μL, clone HIP8, IgG_1, Immunostep, Salamanca, Spain) and a fluorescein isothiocyanate (FITC)-conjugated mouse mAb against the human integrin $\alpha_{IIb}\beta_3$/GPIIb/IIIa (2.5 μL, clone PAC-1, IgM, BD Biosciences), or with an allophycocyanin (APC)-conjugated mAb against human P-selectin (1.25 μL, clone HI62P, IgG_1, Immunostep). The CD41$^+$ population (platelets) was selected according to the gating strategy illustrated in Figure S1 and expressed as percentage of positive platelets.

To determine the grade of leukocyte activation, the expression of CD69 or CD11b was analyzed on circulating neutrophils, monocytes and T lymphocytes. Heparinized whole blood samples were incubated in the dark for 30 min with saturated amounts of a phycoerythrin (PE)-conjugated mAb against human CD69 (clone FN50, IgG_1, Immunostep), an APC-conjugated mouse mAb against human CD69 (clone FN50, IgG_1, BD Biosciences), an APC-conjugated mouse mAb against human integrin CD11b (clone ICRF44, IgG_1, Biolegend, San Diego, CA), or a PE-conjugated mouse mAb against human integrin CD11b (clone CBRM1/5, IgG_1, Biolegend). Fractalkine/CX_3CL1 receptor (CX_3CR1) expression was determined using a PE-conjugated rat mAb against human CX_3CR1 (clone 2A9-1, IgG_{2B}, Biolegend). In some experiments, heparinized blood samples were incubated with EDTA (10 mM, for 15 min at 37ºC) to promote platelet dissociation as described [2]. This disaggregation was measured using the marker CD41 in circulating leukocyte subsets. To do this, heparinized whole blood or EDTA samples were incubated in the dark for 30 min with saturated amounts of a PE/Cy™7-conjugated mouse mAb against human CD41 (clone HIP8, IgG_1, Biolegend) or a CF-Blue™-conjugated mAb against human CD41 (clone HIP8, IgG_1, Immunostep).

Red blood cells were lysed using a commercial lysis buffer (BD FACS™ lysing solution 10× concentrate, BD Biosciences).

The expression of the different markers was determined on $CD16^+$ (neutrophils, Figure S2), $CD14^+$ (monocytes, Table S1, Figure S3), $CD3^+$ (T lymphocytes, Figure S4), $CD8^+$ (cytotoxic lymphocytes, Figure S4), $CD4^+$ (T helper lymphocytes Table S2, Figures S4 and S5) and $CD127^-CD25^+$ (T regulatory lymphocytes, Figure S6) populations. Gating strategies are illustrated in Figures S2–S6.

Quantification of soluble inflammatory and metabolic markers

Heparinized human whole blood (17 IU heparin/mL) was collected and centrifuged to obtain the plasma, which was stored at -80ºC. The following cytokines and chemokines were measured in plasma samples by enzyme-linked immunosorbent assay (ELISA, DuoSet® ELISA Development Systems, R&D Systems, Abingdon, UK) as previously described[3, 4]: human soluble interleukin (IL)-4, IL-6, IL-10, IL-12, tumor necrosis factor (TNF)-α, interferon (IFN)-γ, growth-regulated oncogene-α (GROα/CXCL1), platelet factor-4 (PF-4/CXCL4), IL-8/CXCL8, monocyte chemoattractant protein-1 (MCP-1/CCL2), regulated on activation normal T cell expressed and secreted chemokine (RANTES/CCL5), fractalkine/CX_3CL1, soluble P-selectin (sP-selectin), adiponectin, leptin and ghrelin. Results were expressed as pg or ng/mL of mediator in plasma.

Leukocyte-endothelial cell interactions under flow conditions

Before starting each assay, whole blood was diluted 1:10 with Hank's balanced salt solution (HBSS, Lonza, Barcelona, Spain) without calcium or magnesium at a temperature of at 37ºC. Blood was perfused across HUAEC monolayers, unstimulated or stimulated with 20 ng/mL TNFα (Sigma-Aldrich, Madrid, Spain), for 24 h. Experiments were performed in heparinized blood treated or not with EDTA (10 mM, for 15 min, 37ºC) in order to determine the platelet contribution to leukocyte adhesion [2].

Leukocyte adhesion was determined after 7 min at 0.5 dyn/cm^2. Cells interacting on the surface of the endothelium were visualized and recorded (×20 objective, ×10 eyepiece) using a phase contrast microscopy (Axio Observer A1, Carl Zeiss microscope, Thornwood, NY). For each determination, at least 5 fields were recorded for 10 s and then averaged. Finally, recorded images were saved on a computer for further analysis.

Immunofluorescence studies

To visualize adherent platelet–leukocyte complexes with endothelial cells, we performed an immunofluorescence analysis. Confluent endothelial cells were grown on glass coverslips and stimulated with 20 ng/mL TNFα, for 24 h. Heparinized blood from patients with PH and age-matched controls was incubated without or with EDTA for 1 h. After the flow chamber assay, cells were fixed with 4% paraformaldehyde and blocked in PBS containing 1% BSA. Subsequently, cells were incubated at room temperature for 2 h with an Alexa 488-conjugated antibody against human CD45 (1:50 dilution, green, clone HI30, IgG_1, BioLegend, San Diego, CA) and an APC-conjugated antibody against human CD41 (1:50 dilution, red, clone HIP8, IgG_1, Immunostep, Salamanca, Spain) in 0.1% BSA/PBS. Nuclei from endothelial cells and leukocytes were counterstained with Hoechst (blue, Sigma-Aldrich, Madrid, Spain). Images were captured with Zeis Axio Observer A1 fluorescence microscope.

References

1. Rius C., Piqueras, L., Gonzalez-Navarro, H., Albertos, F., Company, C., Lopez-Gines, C., Ludwig, A., Blanes, J.I., Morcillo, E.J., and Sanz, M.J. Arterial and venous endothelia display differential functional fractalkine (CX3CL1) expression by angiotensin-II. *Arterioscler Thromb Vasc Biol.* **2013**, *33*, 96–104, doi:10.1161/ATVBAHA.112.254870.
2. Postea O., Vasina, E.M., Cauwenberghs, S., Projahn, D., Liehn, E.A., Lievens, D., Theelen, W., Kramp, B.K., Butoi, E.D., Soehnlein, O., Heemskerk, J.W., Ludwig, A., Weber, C., and Koenen, R.R. Contribution of

platelet CX(3)CR1 to platelet-monocyte complex formation and vascular recruitment during hyperlipidemia. *Arterioscler Thromb Vasc Biol.* **2012**, *32*, 1186–1193, doi:10.1161/ATVBAHA.111.243485.
3. Furio E., Garcia-Fuster, M.J., Redon, J., Marques, P., Ortega, R., Sanz, M.J., and Piqueras, L. CX3CR1/CX3CL1 Axis Mediates Platelet-Leukocyte Adhesion to Arterial Endothelium in Younger Patients with a History of Idiopathic Deep Vein Thrombosis. *Thromb Haemost.* **2018**, *118*, 562–571, doi:10.1055/s-0038-1629897.
4. Marques P., Collado, A., Escudero, P., Rius, C., Gonzalez, C., Servera, E., Piqueras, L., and Sanz, M.J. Cigarette Smoke Increases Endothelial CXCL16-Leukocyte CXCR6 Adhesion In Vitro and In Vivo. Potential Consequences in Chronic Obstructive Pulmonary Disease. *Front Immunol.* **2017**, *8*, 1766, doi:10.3389/fimmu.2017.01766.

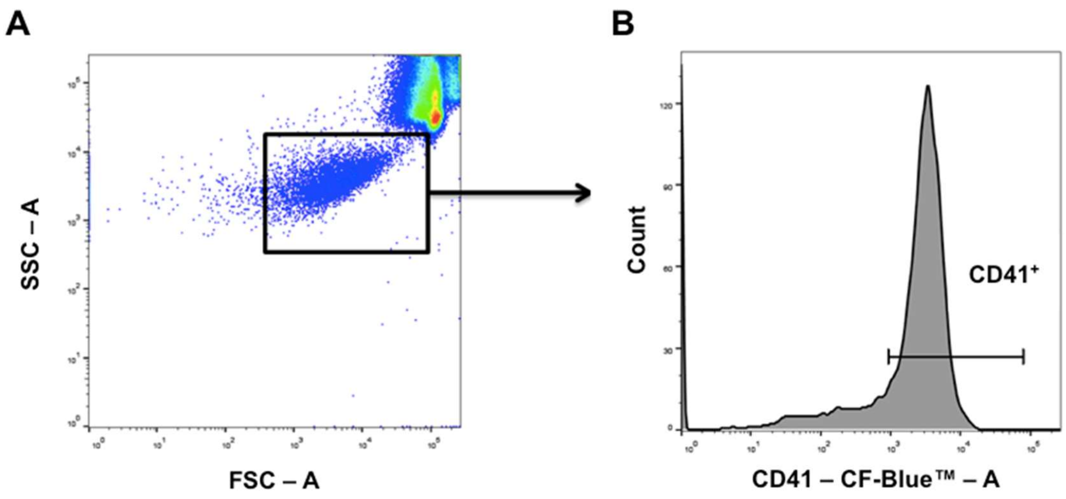

Figure S1. Gating strategy for human platelets in whole blood according to morphological properties and CD41 detection by flow cytometry. Platelets were gated according to a low side scatter (SSC-A) and forward scatter (FSC-A) in a logarithmic scale (**A**) and defined as CD41$^+$ population (**B**).

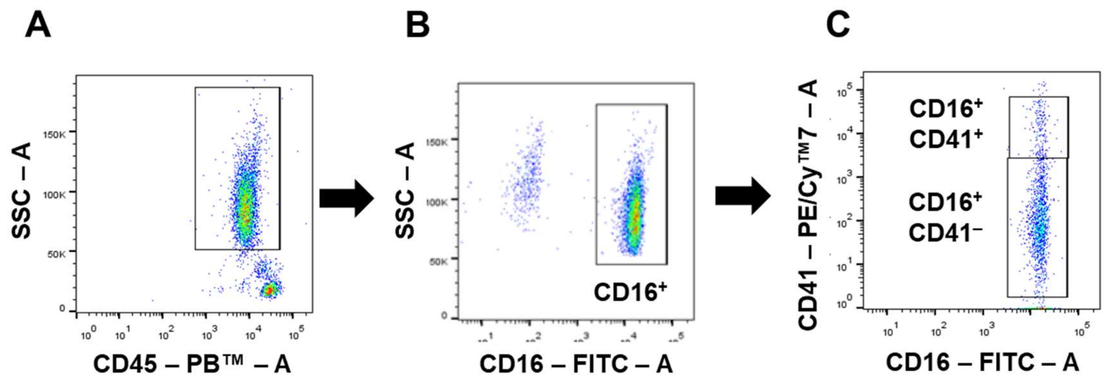

Figure S2. Gating strategy for human neutrophils in whole blood according to morphological properties and CD16 expression by flow cytometry. Populations were selected by CD45 labeling and morphology (high SSC-A, **A**). A CD16 antibody was used to detect neutrophils (CD16$^+$) (**B**). In heparinized blood, neutrophil-platelet-complexes were selected as a CD16$^+$CD41$^+$ population.

Table S1. Differential markers of monocyte subpopulations.

Marker	Cellular Population

CD14$^+$CD16$^-$CCR2$^+$	Monocyte type 1 (Mon 1)
CD14$^+$CD16$^+$CCR2$^+$	Monocyte type 2 (Mon 2)
CD14$^+$CD16$^+$CCR2$^-$	Monocyte type 3 (Mon 3)

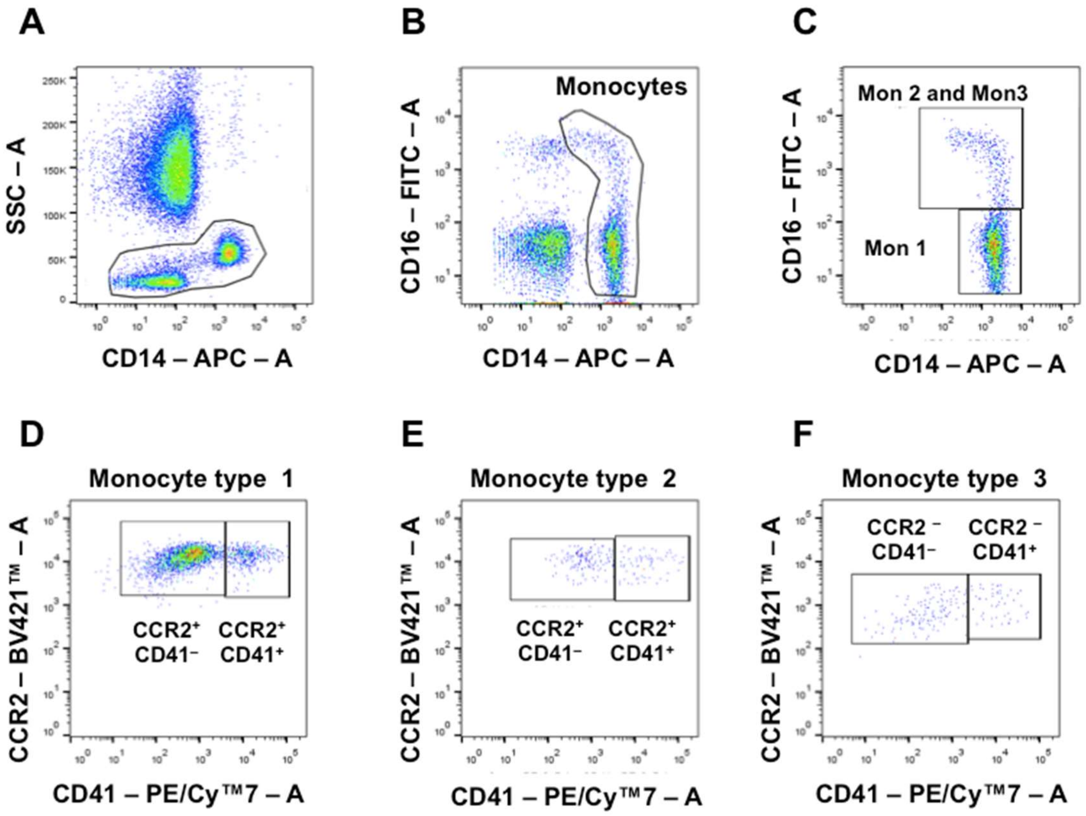

Figure S3. Gating strategy for human monocyte detection in whole blood by flow cytometry. Monocytes were selected by CD14 labeling and morphology (medium SSC-A, **A**). For the detection of monocyte subpopulations, CD16 and CCR2 markers were used (**B**–**F**). Monocytes-platelets complexes were selected as CD14$^+$CD41$^+$ populations in heparinized whole blood, and platelet-free monocytes were gated as CD14$^+$CD41$^-$ from blood incubated with EDTA (**D** and **E**).

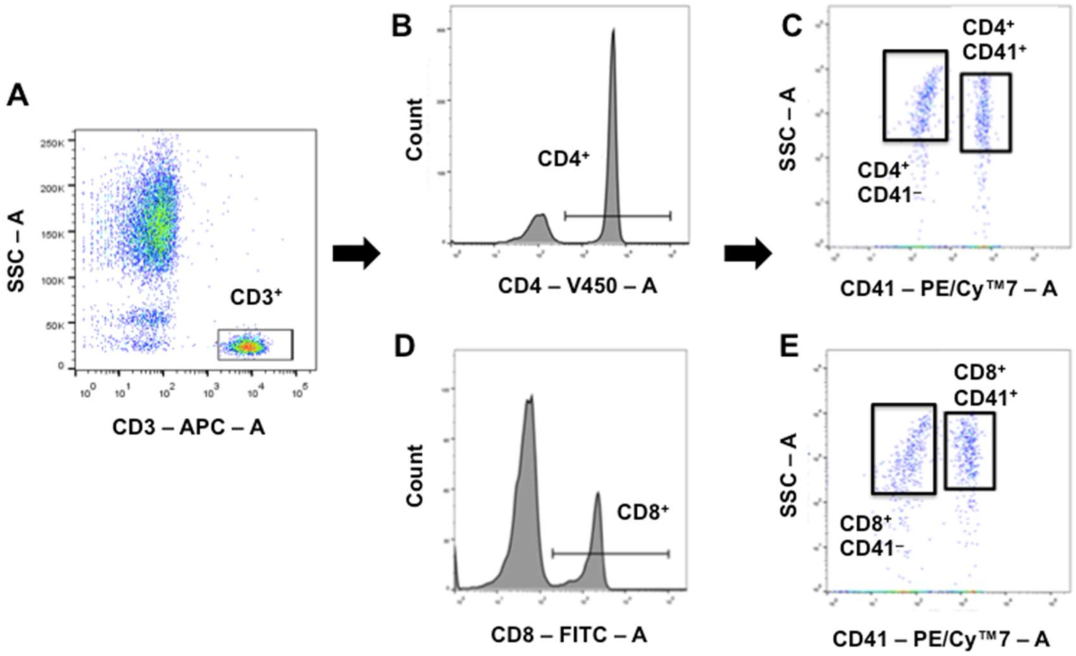

Figure S4. Gating strategy for human T lymphocyte detection in whole blood by flow cytometry. T lymphocytes were selected as a $CD3^+$ population and with a low SSC-A (**A**). T helper (Th) lymphocytes were selected as the $CD3^+CD4^+$ population (**B**). In heparinized blood, Th lymphocyte-platelet complexes were selected as the $CD3^+CD4^+CD41^+$ population, whereas platelet-free Th lymphocytes were gated as $CD3^+CD4^+CD41^-$ from blood incubated with EDTA (**C**). Cytotoxic lymphocytes were selected as $CD3^+CD8^+$ (**D**). In heparinized blood, cytotoxic lymphocyte-platelet complexes were selected as the $CD3^+CD8^+CD41^+$ population (**E**).

Table S2. Differential markers for detection of Th lymphocyte subpopulations.

Marker	Cellular Population
$CXCR3^+CCR6^-$	Type 1 T helper (Th1)
$CXCR3^-CCR6^-$	Type 2 T helper (Th2)
$CXCR3^+CCR6^+$	Type 17 T helper (Th17)

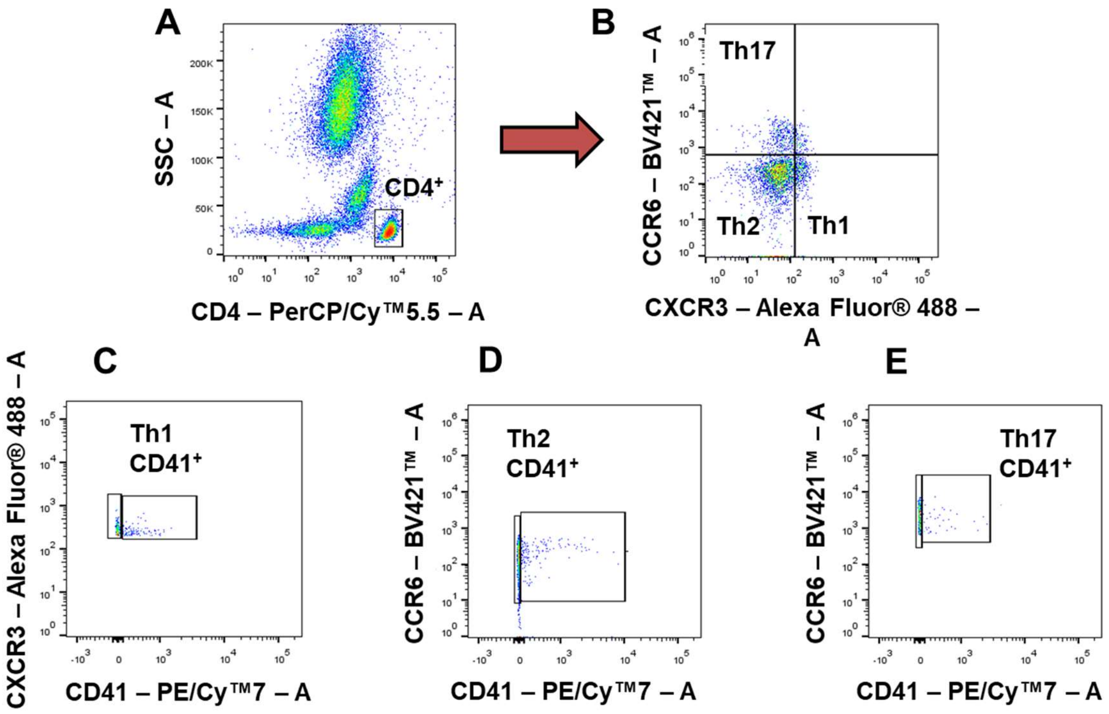

Figure S5. Gating strategy for human T helper lymphocyte detection in whole blood by flow cytometry. T helper (Th) lymphocytes were selected as the $CD4^+$ population and with a low SSC-A (**A**). Th lymphocyte subpopulations were detected with the markers CXCR3 and CCR6 **(B)**. In heparinized blood, Th1 lymphocyte-platelet complexes were selected as $CXCR3^+CCR6^-CD41^+$ (**B** and **C**). Th2 lymphocyte-platelet complexes were selected as $CXCR3^-CCR6^-CD41^+$ (**B** and **D**), and Th17 lymphocyte-platelet complexes were selected as $CXCR3^+CCR6^+CD41^+$ (**B** and **E**).

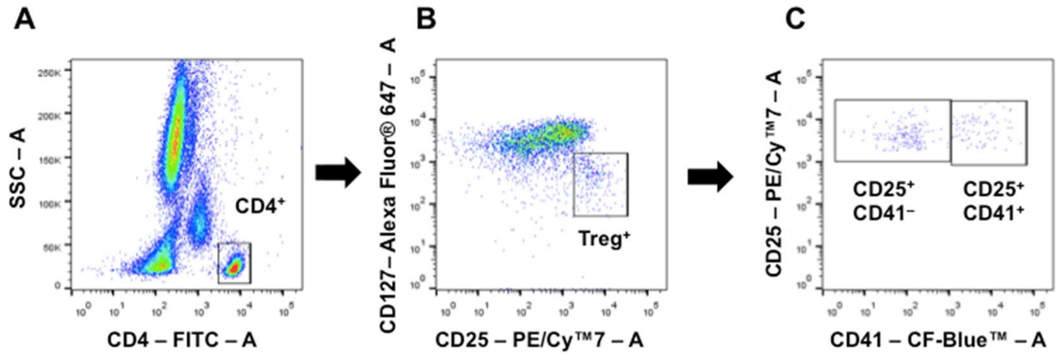

Figure S6. Gating strategy for human regulatory T lymphocyte (Treg) detection in whole blood by flow cytometry. Treg lymphocytes were selected as the $CD4^+$ population and with a low SSC-A (**A**). Treg lymphocytes were detected with the markers CD127 and CD25 **(B)**. Treg lymphocyte-platelet complexes were selected as the $CD127^-CD25^+CD41^+$ population from heparinized whole blood (**C**).

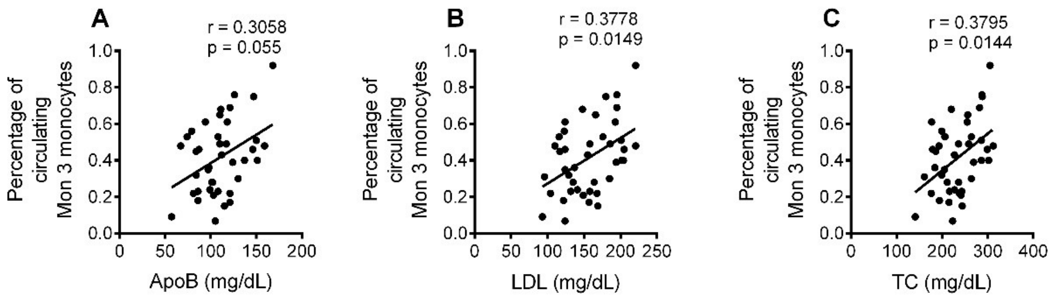

Figure S7. Positive correlation between the percentage of nonclassical/Mon 3 monocytes and the circulating levels of ApoB (**A**), LDL (**B**) and TC (**C**). (n = 21 control subjects and n = 22 PH patients).
